# Consuming Transgenic Goats' Milk Containing the Antimicrobial Protein Lysozyme Helps Resolve Diarrhea in Young Pigs

**DOI:** 10.1371/journal.pone.0058409

**Published:** 2013-03-13

**Authors:** Caitlin A. Cooper, Lydia C. Garas Klobas, Elizabeth A. Maga, James D. Murray

**Affiliations:** 1 Department of Animal Science, University of California Davis, Davis, California, United States of America; 2 Department of Population Health and Reproduction, University of California Davis, Davis, California, United States of America; University of Padova, Italy

## Abstract

Childhood diarrhea is a significant problem in many developing countries and *E. coli* is a main causative agent of diarrhea in young children. Lysozyme is an antimicrobial protein highly expressed in human milk, but not ruminant milk, and is thought to help protect breastfeeding children against diarrheal diseases. We hypothesized that consumption of milk from transgenic goats which produce human lysozyme (hLZ-milk) in their milk would accelerate recovery from bacterial-induced diarrhea. Young pigs were used as a model for children and infected with enterotoxigenic *E. coli*. Once clinical signs of diarrhea developed, pigs were fed hLZ-milk or non-transgenic control goat milk three times a day for two days. Clinical observations and complete blood counts (CBC) were performed. Animals were euthanized and samples collected to assess differences in histology, cytokine expression and bacterial translocation into the mesenteric lymph node. Pigs consuming hLZ-milk recovered from clinical signs of infection faster than pigs consuming control milk, with significantly improved fecal consistency (*p* = 0.0190) and activity level (*p* = 0.0350). The CBC analysis showed circulating monocytes (*p* = 0.0413), neutrophils (*p* = 0.0219), and lymphocytes (*p* = 0.0222) returned faster to pre-infection proportions in hLZ-milk fed pigs, while control-fed pigs had significantly higher hematocrit (*p* = 0.027), indicating continuing dehydration. In the ileum, pigs fed hLZ-milk had significantly lower expression of pro-inflammatory cytokine IL-8 (*p* = 0.0271), longer intestinal villi (*p*<0.0001), deeper crypts (*p* = 0.0053), and a thinner lamina propria (*p* = 0.0004). These data demonstrate that consumption of hLZ-milk helped pigs recover from infection faster, making hLZ-milk an effective treatment of *E. coli*-induced diarrhea.

## Introduction

Since the arrival of genetic engineering scientists have foreshadowed the use of transgenic animals for improving agriculture [1], as transgenic technology allows for specific genetic changes to be made to improve animals for agriculture that cannot be achieved using traditional animal breeding approaches. Strategies were proposed to generate transgenic dairy animals producing altered milk proteins to improve functional characteristics of the milk [2]. As early as 1988 human lysozyme was identified as a potential candidate protein for production in the milk of dairy animals. Due to the antimicrobial and cationic properties of lysozyme it was theorized that transgenic milk containing lysozyme may alter milk processing properties, improve udder health, and prevent infections [3]. In 1999 the Artemis line of transgenic goats producing milk containing human lysozyme (hLZ-milk) was established [4]. Human lysozyme was chosen to avoid any adverse reactions humans may have to consumption of other forms of lysozyme, and human lysozyme is a more active antimicrobial than other forms of lysozyme such as hen egg white lysozyme (HEWL) [5]. Goats were chosen because they have a shorter generation interval than cows, are adapted to thrive in a wide range of environments, and are already a key part of the agricultural system in many developing parts of the world. Since the establishment of the Artemis line, numerous studies on the health and welfare of the transgenic goats [6], [7] and the effects of consumption of the hLZ-milk, by both goats and pigs, have been reported [8]–[11]. However, this is the first report that demonstrates that the hLZ-milk is an effective treatment for diarrhea caused by a bacterial infection in the gastrointestinal (GI) tract, fulfilling the promise of using transgenic animals to produce food with improved functional characteristics. This finding has numerous implications for human health, particularly in developing nations.

Over a million children die each year from diarrheal diseases caused by bacterial and viral infections; with pathogenic strains of *E. coli* being one of the main causative agents [12]. During a GI tract infection bacteria release toxins and the body mounts an inflammatory response which recruits immune cells to help clear the bacteria. The toxins and inflammation cause breakdown of the tight junctional complexes between the intestinal epithelial cells and disrupt ion channel regulation, which together lead to an influx of water and ions into the intestinal lumen, causing diarrhea [13]. The effects of diarrhea include dehydration, electrolyte imbalance, and malnutrition due to decreased nutrient absorption. Persistent diarrhea in children that results in malnutrition can be particularly detrimental to development, leaving children with mental and growth deficiencies that can last a lifetime [12].

The protein lysozyme is an important non-specific antimicrobial factor found in many bodily secretions. HEWL has been used for many years as a food additive and numerous studies have been conducted on the safety and effects of lysozyme. The FDA ruled HEWL as a generally recognized as safe (GRAS) compound in 1998 [14], and the JEFCA-Joint FAO/WHO Expert Committee on Food Additives approved its use as a food additive [15]. High quantities of lysozyme are found in human breast milk, but there is little in the milk of ruminant animals such as cows and goats [16]. Lysozyme acts as a 1,4-β-acetylmuramidase that hydrolyzes the glycosidic bond between *N*-acetylmuramic acid and *N*-acetyl-glucosamine in the peptidoglycan layer of bacterial cell walls [17], and has significant immunomodulatory effects on neutrophils, lessening the chemotactic movement of neutrophils towards *E. coli* bacterial supernatant [18], [19]. Breastfed infant’s experience health benefits that include a lower incidence of GI illnesses compared to formula-fed infants [20], which is in part attributed to lysozyme’s action against a variety of microbes and its ability to inhibit systemic inflammation [21].

Lysozyme in human milk contributes to the establishment of stable bacterial populations within the gut, favoring the growth of beneficial bacteria such as *Lactobacillus* and *Bifidobacteria* [22], and in experimental animal models purified lysozyme has been shown to lower levels of pathogenic strains of *E. coli* [23]. The epithelium of the intestinal villi must accomplish the conflicting functions of absorbing nutrients and acting as a barrier against luminal bacteria, thus changes in the host microbiota affect the morphology of the intestinal villi. Some bacteria and their metabolites can promote stabilization of tight junctions [24], increase villi height and crypt depth [25], while strains of pathogenic *E. coli* can damage the intestinal epithelium and decrease villi height [26].

Many developing parts of the world rely on livestock as a main source of food, and through genetic engineering we can provide agriculturally relevant animals with novel traits targeted to help solve the problems facing these developing areas. As previously mentioned, we have generated a herd of transgenic goats that produce hLZ-milk [27]. The lysozyme concentration of the transgenic goats’ hLZ- milk is 270 mg/L, which is 68% of that found in human milk, and the hLZ-milk has been shown to significantly lower levels of *E. coli* in both *in vitro* and *in vivo* studies [6]–[9]. Pigs have very similar GI physiology to humans and on average there is a moderate amount of lysozyme (10 mg/L) in pig’s milk [28]. Using pigs as an animal model for human health, consumption of pasteurized hLZ-milk by young healthy pigs positively impacted GI morphology, serum metabolites, lymphocyte populations, increased expression of an anti-inflammatory cytokine, and enriched the fecal microbial populations of bacteria associated with gut health including *Lactobacillus* and *Bifidobacteria*. Also, over the course of multiple feeding trials no adverse effects were observed in pigs consuming hLZ-milk [9]–[11], [29]. Given the positive systemic and intestinal health effects observed in healthy animals after consumption of hLZ-milk, as well as lysozyme’s innate antimicrobial properties, goats’ milk containing hLZ was tested as a treatment for enterotoxigenic *E.coli* (ETEC)-induced diarrhea using a porcine model.

## Materials and Methods

### Milk

Milk was acquired from the UC-Davis goat facility. The hLZ-milk was collected and pooled from lactating does from the Artemis line of goats, while control goat milk was collected and pooled from non-transgenic control does. Milk was pasteurized at 73.8°C then immediately cooled and stored at 4°C for no more than a week before being fed to the pigs.

### Animal Care and Ethics

Six -week-old male Hampshire Yorkshire crossbred pigs, from a specific pathogen free facility were obtained. Pigs were genotyped for F4ab/ac which indicates early (<24 hours) or later (>24 hours) onset of *E. coli* infections and all pigs were homozygous for later onset [30]. Pigs were kept in a temperature-controlled room between 25°C and 27°C with ad libitum access to food and water for the duration of the trials. The specific diet and rearing of the pigs used for this study has been previously described [9]. The diet included 0.0005% of Lincomix (Pfizer Animal Health, New York, NY) as an antibiotic growth promoter. Upon arrival pigs were randomly assigned to either the hLZ-milk or control milk group. All procedures performed as well as the care and use of the animals in this study was approved by the UC Davis Institutional Animal Care and Use Committee (protocol number 15741), under AAALAC approved conditions.

### Bacterial Culture and Preparation

The O149:F4+ ETEC strain (Escherichia coli Laboratory, University of Montréal: 864075856716) was provided by Dr. John Fairbrother. The bacteria were cultured in a level 2 biosafety lab. One liter of TSB was inoculated and incubated at 37°C for 24 hours. Samples of inoculum were taken, diluted in PBS, and spiral plated onto LB plates for colony remuneration. The inoculum was then centrifuged at 4000 RPM for 10 minutes to isolate bacteria. The supernatant was removed and the bacteria were washed with PBS and spun again at 4000 RPM for 10 minutes. Supernatant was removed and the bacteria were suspended in saline solution. Pigs were orally dosed with bacteria suspended in saline solution.

### Dosing Schedule for Bacteria and Milk

**Figure 1:**
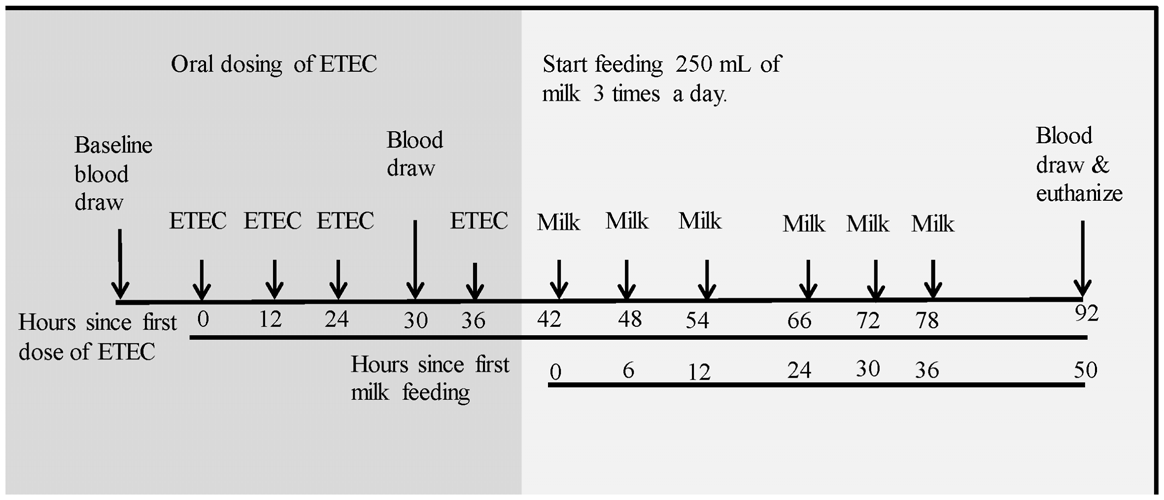
Time line of experiment. Timeline includes dosing of ETEC, blood draws, and milk feeding schedule.

A preliminary study was done to determine the dose of ETEC needed to cause diarrhea as well as monitor the progression of the clinical symptoms over time. Eight pigs were orally dosed with 2.5×10^∧10^ colony forming units (CFUs) of the porcine specific O149:F4+ ETEC. A total of four doses were given, once every 12 hours. This dosing regimen consistently caused sustained diarrhea in all pigs by 30 hours after the first dose, and by 72 hours after the first dose most pigs were starting to show signs of recovery. With this optimized dosing protocol two replicate milk treatment trials were conducted with 10 and 12 pigs respectively. Starting six hours after the final dose of ETEC was administered (42 hours), according to the groups they were previously assigned to, pigs were fed 250 mL of either pasteurized control goats’ milk or hLZ-milk three times daily for two days. Pigs were euthanized 92 hours after the first dose of ETEC (Figure 1).

### Blood Samples

Blood was collected from the anterior vena cava into EDTA coated vacutainer tubes (Becton Dickinson Company, Franklin Lakes, NJ) before infection, 30 hours after the first dose of ETEC, and at the end of the study (92 hours after the first dose of ETEC). Complete blood count analyses on the samples were performed by Idexx Laboratories using an ADVIA 120 Hematology System (Siemens Healthcare Diagnostics Inc., Tarrytown, NY). A total of seventeen parameters were measured.

### Clinical Observations

The fecal consistency and activity level of the pigs were assessed and recorded every twelve hours for the duration of the trial and feces were removed from the pens after each assessment. The same person, who was blinded to the treatments, conducted both observations. The scores were on a scale from zero to four. For fecal consistency a score of 4 indicated normal, solid feces, while a score of 0 was very watery feces. Similarly for activity scores a score of 4 was assigned to an alert and responsive pig that would react quickly to a person and to being fed while a score of 0 indicated a pig that was very lethargic and would refuse to move in response to being fed.

### Necropsy and Sample Collection

Pigs were euthanized using pentobarbital sodium (Fatal-Plus®, Vortech Pharmaceuticals, Ltd.) and tissue samples were collected. Duodenum samples were taken 20 cm below the pyloric sphincter and ileum samples were taken 20 cm above the ileocecal junction. Samples were also taken from the ileum mesenteric lymph node (MLN).

### Histology

Samples from the duodenum and ileum were fixed in paraformaldehyde for 48 hours, then in 70% ethanol overnight. Samples underwent a process of step wise dehydration and were injected with paraffin (Tissue-Tek VIP 4 Tissue Processor, Sakura Int.). Paraffin injected samples were then embedded into paraffin to create blocks, which were subsequently cut into 6 µm sections and mounted onto slides. Slides were then stained with hematoxylin and eosin. Slides were analyzed by photographing then measuring the villi height, width, lamina propria thickness, and crypt depth at 10X magnification, using Spot Advanced Software (v3.4, Diagnostic Instruments). In addition, the number of lymphocytes and goblet cells per villus were counted at 40X magnification and analyzed as cells per unit villous height. At least five villi were measured per slide.

### Total RNA Extraction

Total RNA was extracted from duodenum and ileum sections using Trizol reagent (Invitrogen, Carlsbad, CA) and was eluted in an appropriate amount of RNase-free water (Qiagen, Valencia, CA). For each sample, the integrity of the extracted RNA was analyzed by agarose gel electrophoresis by staining with ethidium bromide and visualization under UV light. Following extraction, RNA was treated with RQ1 DNase I (Promega, Madison WI) to remove any DNA contamination. Then the concentration was quantified and purity determined (OD260/OD280 absorption ratio >1.9) using a NanoDrop® ND-2000C Spectrophotometer (NanoDrop Technologies Inc., Wilmington, DE). For each sample, 1 µg of RNA was converted to cDNA using OligoDT, 59 RT Buffer (Invitrogen), 0.1 M DTT,RNasin, dNTPs, DEPC dH20 and SuperScriptII reverse transcriptase (Invitrogen, Carlsbad, CA).

### Quantitative Real-time RT-PCR

Quantitative real-time RT-PCR was performed using a 7500Fast Real-Time PCR System (Applied Biosystems, Foster City, CA). Each reaction contained 12.5 µl of Fast SYBR Green (Applied Biosystems, Foster City, CA, USA) probe master mix, 1 µl of 10 µM reverses primer, 1 µl of 10 µM forward primer, 50 ng cDNA and dH20 in a total sample volume of 25 µl. Standard curves for each gene were constructed using cDNA template consisting of pooled cDNA from different pigs at concentrations of 300 ng/reaction with 1∶2 dilutions down to 4.68 ng/reaction. Amplification consisted of 3 steps: denaturation for 15 s at 94C°, annealing for 30 s at 60C°, and product extension for 30 s at 72C°. Fluorescence data was collected on the annealing step. This was repeated for a total of 40 cycles. Then product dissociation was measured in 3 steps: 95C° for 15 s, 60C° for 1 min, and 95C° for 15 s. For each plate, threshold and baseline were individually set and CT values determined using the 7500 Fast software. 40 cycles of qPCR at 95°C of denaturing for 15 s and 60°C of annealing/extension for 1 min. All reactions were conducted in triplicate. All primers were porcine specific and validated before use (Table 1).

**Table 1:** Porcine specific primer sequences for qRT-PCR.

Gene name	Forward primer (5′-3′)	Reverse Primer (5′-3′)	Product (bp)	Acquisition number
β-actin	GGATGCAGAAGGAGATCACG	ATCTGCTGGAAGGTGGACAG	130	U07786
IL-8	TGGCAGTTTTCCTGCTTTCT	CAGTGGGGTCCACTCTCAAT	154	M86923
IFN-γ	CCATTCAAAGGAGCATGGAT	GAGTTCACTGATGGCTTTGC	146	AY188090
TNF-α	CACCACGCTCTTCTGCCTACTGC	CCTCGGCTTTGACATTGGCTAC	164	X57321
IL-17	TCATGATCCCACAAAGTCCA	AGTCCATGGTGAGGTGAAGC	146	NM_001005729
IL-10	TGATGGGGAGGATATCAAGG	TGGAGCTTGCTAAAGGCACT	150	NM_214041
TGF-β	CGAGCCCTGGATACCAACTA	AGGCTCCAGATGTAGGGACA	164	Y00111
Foxp3	CTGACAAGGGTTCCTGCTGT	GAAATCTGGGAACGTGCTGT	149	NM_001128438

### Bacterial Translocation

Samples from the mesenteric lymph nodes were washed in sterile PBS. A 100 mg piece was weighed and homogenized. The homogenate was diluted in sterile PBS and plated on sheep blood agar plates. Plates were allowed to incubate at 37°C for 48 hours at which time the number of total and hemolytic colonies were counted.

### Statistical Analysis

Statistical evaluations for hematological, histological, observational, and bacterial data were conducted by use of the SAS statistical software, Version 9.3 (SAS Inc.,Cary, NC). Hematological, histological, and bacterial data were compared using the parametric test one-way analysis of variance (ANOVA) and differences were tested using the Dunnett's test. Observation data was compared using the repeated measures function in SAS and the Greenhouse-Geisser Epsilon test to determine differences. Statistical analysis for fold expression differences from the qPCR assay was performed using REST-MCS software using β-actin as a housekeeping gene. Differences were determined using the Pair Wise Fixed Reallocation Randomisation Test [31]. For all tests p-values <0.05 were considered statistically significant.

## Results

### Clincal Observation

**Figure 2:**
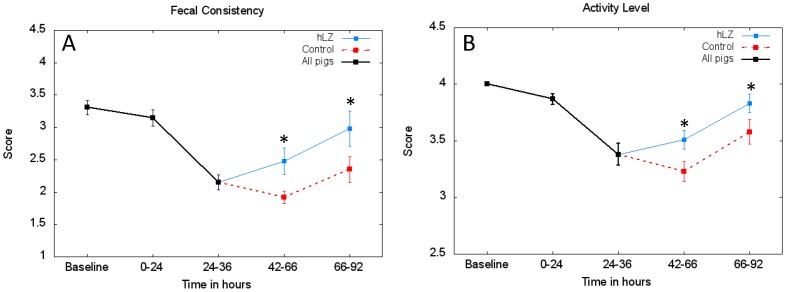
Pigs fed hLZ- milk have improved fecal consistency and activity scores. Average scores (mean± SEM) for (A) fecal consistency and (B) activity level for the entire experiment. When analyzed from the start of milk treatments to the end of the study, pigs fed hLZ-milk (n = 10) had improved fecal consistency compared to pigs fed control milk (n = 12) (*p* = 0.0090), as well as an increased level of activity (*p* = 0.0350). Data was analyzed using the repeated measures function in SAS and the Greenhouse-Geisser Epsilon method to calculate *p* values *indicates *p*<0.05.

Clinical signs of the illness became apparent in all pigs between 24 to 36 hours after the first dose of ETEC as the average fecal consistency and activity scores dropped, but once milk treatments started until the end of the study, pigs fed hLZ-containing milk had significantly higher fecal consistency (*p* = 0.0190) and were significantly more active (*p* = 0.0350) compared to pigs fed control milk (Figure 2). By the end of the study, both the average fecal consistency and activity scores for pigs fed hLZ-milk were approaching the pre-infection averages.

### Complete Blood Count Analysis

**Figure 3:**
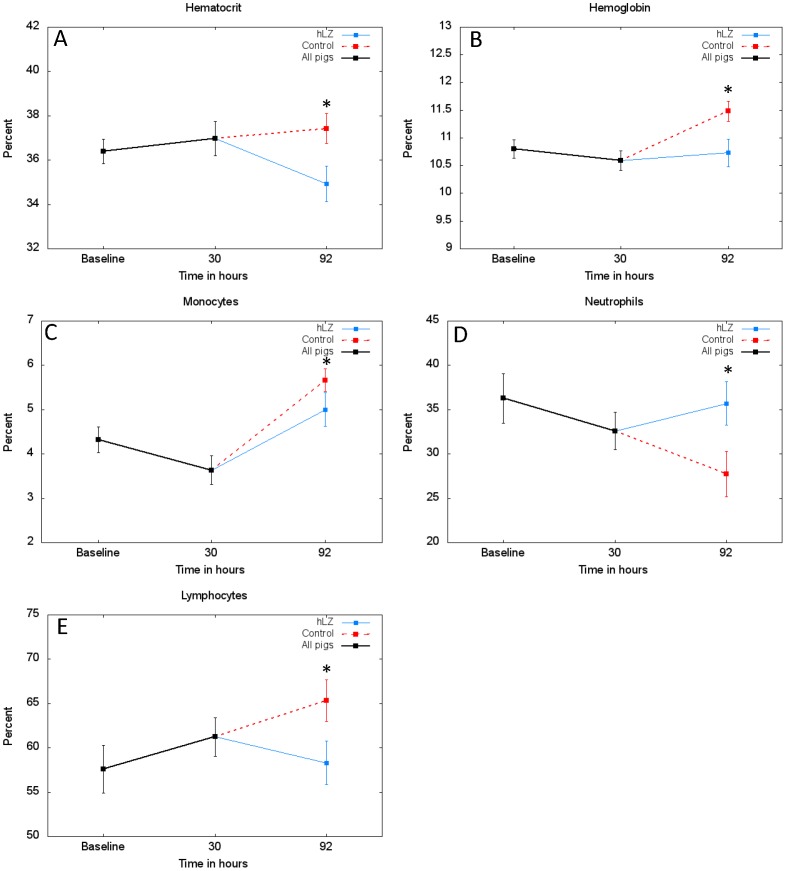
Pigs fed hLZ-milk are less dehydrated and clear the ETEC infection faster than control-fed pigs. Concentrations (mean ± SEM) of (**A**) hematocrit (**B**) hemoglobin (**C**) monocytes (**D**) neutrophils and (**E**) lymphocytes over the course of the study as determined by CBC analysis. Dunnett’s test was used for single comparisons between the hLZ-milk fed (n = 10) and control milk fed (n = 12) groups, *indicates *p*<0.05. Pigs fed hLZ-milk suffer from less dehydration (significantly lower concentrations of hematocrit and hemoglobin) and return to baselines levels of circulating leukocytes (significantly fewer monocytes and lymphocytes, and significantly more neutrophils in circulation) compared to control-fed pigs.

At the end of the study, pigs fed control milk were significantly more dehydrated than animals fed hLZ-milk. Control-fed pigs had significantly higher hematocrit (*p* = 0.027) and hemoglobin concentrations (*p* = 0.0251), compared to pigs fed hLZ-milk (Figures 3a and 3b). Pigs fed hLZ-milk had significantly lower proportion of circulating monocytes (*p* = 0.0413) at the end point of the study (Figure 3c). Infection caused the proportion of neutrophils to drop in all pigs and in pigs fed control milk neutrophils continued to decrease, however in pigs fed hLZ-milk the percentage of neutrophils was significantly higher than in control pigs (*p* = 0.0219), and had returned to pre-infection proportions at the end point of the trial (92 hours) (Figure 3d). At 92 hours, pigs fed hLZ-milk also demonstrated significantly less recruitment of lymphocytes from peripheral lymphoid organs into circulation (*p* = 0.0222) (Figure 3e).

### Morphological Changes of Villi in the Duodenum and Ileum

**Figure 4:**
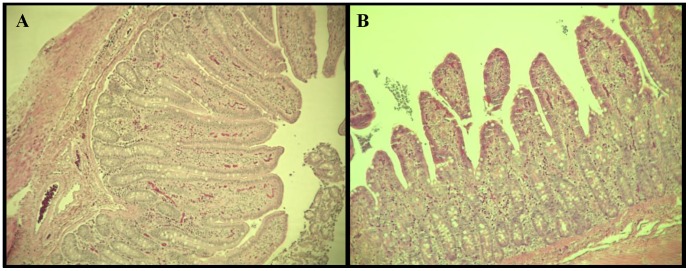
Pigs fed hLZ-milk had less ETEC-induced intestinal damage at the end of the experiment. Histological sections at 10X magnification showing average villi from the ileum of pigs (**A**) fed hLZ-milk or (**B**) control milk. Pigs fed hLZ-milk had reduced damage due to ETEC infection as seen by increased surface area (taller villi) and less inflammation (thinner lamina propria), compared to pigs fed control milk.

In both the duodenum and ileum, pigs fed hLZ-milk had significantly taller villi than those fed control milk (*p* = 0.0103, *p*<0.0001, respectively). In the duodenum, pigs fed hLZ-milk also tended to have fewer intraepithelial leukocytes per micron of villi height compared to control-fed pigs (*p* = 0.099), while in both the duodenum and ileum numbers of goblet cells were not significantly different between hLZ-milk and control-milk fed animals. In the ileum, the villi of hLZ-milk fed pigs tended to be thinner (*p* = 0.058), with significantly deeper crypts (*p* = 0.0053). The pigs fed hLZ-milk had a significantly thinner lamina propria layer (*p* = 0.0004) compared to control milk-fed pigs, indicating less inflammation (Table 2, Figure 4).

**Table 2:** Measurements† from H&E stained slides from the duodenum and ileum of hLZ-fed (*n* = 10) and control-fed animals (*n = 12)*.

	Duodenum		
	Control milk	hLZ-milk	*p* value
**Villi height (µm)**	429.92±150.07	493.67±143.34	**0.0103** *
**Villi width (µm)**	150.00±38.90	164.79±55.75	0.2062
**Crypt Depth (µm)**	117.39±63.77	129.82±63.58	0.2825
**Lamina Propria (µm)**	476.63±173.78	434.06±145.87	0.1307
**Lymphocytes/unit height (µm)**	0.1615±0.0641	0.1402±0.0788	0.0990
**Goblet cells/unit height (µm)**	0.0289±0.0236	0.0335±0.0177	0.4862
	**Ileum**		
	**Control milk**	**hLZ-milk**	***p*** ** value**
**Villi height (µm)**	431.76±136.41	560.10±112.72	**<0.0001** *
**Villi width (µm)**	166.48±128.49	135.91±41.14	0.0580
**Crypt Depth (µm)**	115.26±74.56	154.32±89.99	**0.0053** *
**Lamina Propria (µm)**	268.30±167.92	180.51±111.28	**0.0004** *
**Lymphocytes/unit height (µm)**	0.0997±0.0406	0.1078±0.0549	0.5956
**Goblet cells/unit height (µm)**	0.0288±0.0134	0.0322±0.0138	0.2811

† Measurements presented as mean ± S.D.

* Indicates significant differences between control-milk and hLZ-milk groups, *p*<0.05.

### Transcription of IL-8 in the Ileum

At 92 hours after the first dose of ETEC, expression of the pro-inflammatory cytokine IL-8 was significantly lower in the ileum of pigs fed hLZ-milk compared to those fed control milk (*p = *0.0271). Overall there was a 0.905 fold decrease in IL-8 transcription in pigs fed hLZ-milk, in comparison to pigs fed control milk. There were no statistically significant differences in any of the other cytokines tested (Table 3).

**Table 3:** Expression levels of cytokines in the duodenum and ileum of hLZ-fed (*n* = 10) and control-fed animals (*n = 12)*.

Gene name	Area of the gut	B-actin adjusted CT value		*P* value	Fold change in expression
		Control milk	hLZ-milk		
IL-8	Duodenum	32.74	32.94	0.226	NA
	Ileum	32.51	33.66	0.027*	**.905 ↓**
IFN-γ	Duodenum	32.33	32.38	0.670	NA
	Ileum	32.46	32.32	0.256	NA
TNF-α	Duodenum	32.55	32.65	0.269	NA
	Ileum	32.38	32.60	0.715	NA
IL-17	Duodenum	33.28	33.73	0.606	NA
	Ileum	33.32	33.76	0.617	NA
IL-10	Duodenum	32.46	32.87	0.541	NA
	Ileum	33.61	34.02	0.705	NA
TGF-β	Duodenum	35.60	36.01	0.839	NA
	Ileum	35.59	36.09	0.812	NA
Foxp3	Duodenum	29.55	29.47	0.430	NA
	Ileum	28.33	28.51	0.834	NA

* Indicates significant differences between control-milk and hLZ-milk groups, *p*<0.05.

### Bacterial Translocation to the Mesenteric Lymph Node

To assess the integrity of the intestinal epithelial cell tight junctions and quantify the amount of bacteria that migrated into the MLN, a bacterial translocation assay was conducted [33]. No statistically significant differences were seen in numbers of hemolytic or total bacteria. Pigs fed control milk had an average of 9.439±1.399 log (CFUs/gram)±S.D. total bacteria, while pigs fed hLZ-milk had 8.538±1.122 log (CFU/gram) ±S.D. total bacteria (*p* = 0.1194). For hemolytic bacteria control fed pigs had an average of 4.089±0.671 log (CFUs/gram)±S.D and hLZ-milk fed pigs had an average of 3.597±0.583 log (CFUs/gram)±S.D (*p = *0.158).

## Discussion

Based on the results of these assays, we conclude that hLZ-milk is an effective treatment for a diarrhea caused by ETEC infection. This is supported by clinical observations, hematological, histological, and transcriptional data demonstrating that the ETEC infection was resolved more rapidly in pigs fed hLZ-milk in comparison to pigs fed control milk. Specifically, clinical observations showed that pigs fed with control milk continued to have watery feces, which was confirmed by the CBC analysis showing that control milk fed pigs had higher concentrations of hematocrit and hemoglobin, which are both indicative of dehydration. Clinical observations showed that control-fed pigs were also less active, another common symptom of both an acute phase response to infection and to dehydration.

Diarrhea is caused by the influx of water and ions into the intestinal lumen, and this persistent water loss causes dehydration. This influx is caused by disruption of ion transporters [34] and the breakdown of tight junctions that hold together the intestinal epithelial cells in response to phosphorylation of myosin light chain kinase (MLCK) [35]. The breakdown of the tight junctions allows responding leukocytes to travel easily from circulation through the tissue and to the site of infection [36]; however it also allows for paracellular movement of water into the intestinal lumen [13].

The CBC analysis revealed that feeding hLZ-milk significantly decreased the demand for leukocytes, specifically monocytes, lymphocytes, and neutrophils at the site of infection. Splenic monocytes are recruited into circulation during an infection and home to the foci of inflammation. Thus, monocytemia is indicative of the acute phase of bacterial infection. Neutrophils are also quickly recruited to the site of a bacterial infection through chemotaxis, in response to pro-inflammatory cytokines such as IL-8 and INF-γ. Pigs do not store neutrophils thus there is a small pool of circulating neutrophils that are recruited out of circulation during an infection that can only be replaced by newly produced neutrophils from hematopoietic cells in the bone marrow. As an infection is cleared, the demand for neutrophils decreases, thus less are recruited from circulation, allowing circulating proportions to return to normal, however if an infection persists neutrophils will continue to decrease.

Lymphocytes are a key cell type involved in directing and propagating the adaptive immune response. After maturation they reside in the lymph nodes, spleen, and other lymphatic tissue. Circulating lymphocytes increased over the course of the infection and continued to rise in pigs fed control milk, while in pigs fed hLZ-milk the proportion of circulating lymphocytes returned closer to baseline amounts, indicating that pigs fed hLZ-milk had less demand for lymphocytes.

The pro-inflammatory cytokine IL-8 attracts macrophages, neutrophils, polymorphonuclear leukocytes (PMNs), and phagocytes as a first step in recruitment and induction of an inflammatory response [32]. IL-8 causes chemotaxis of leukocytes from peripheral lymphoid organs to the site of infection, and pigs fed hLZ-milk had significantly lower expression of IL-8 in the ileum than pigs fed control milk. This is also supported by the histological data, which showed fewer leukocytes in the epithelial layer of the duodenum of hLZ-milk fed pigs.

During an inflammatory reaction in the intestine the lamina propria is especially responsive to inflammation. In the ileum, the lamina propria of control fed pigs was significantly thicker, indicating continued inflammation [37]. In the ileum, the control milk-fed pigs had short, wide villi, a sign that the villi had been damaged and were still in the process of repairing themselves. Shorter villi damaged by infection provide less surface area for absorption [26], further exacerbating any nutritional deficiencies caused by the infection and subsequent diarrhea. The longer villi in the hLZ-milk fed pigs indicated less damage to the villi due to faster clearing of the pathogenic bacteria. The hLZ-milk fed pigs also had deeper crypts, which contain the multicomponent local stem cells that give rise to terminally differentiated functional cells including enterocytes [38]. Previous studies show during intestinal inflammation increased crypt depth is closely correlated with increased crypt cell proliferation and improved intestinal recovery [39].

Overall, pigs that were fed hLZ-milk as a treatment for an intestinal ETEC infection recovered faster than pigs fed control milk. They were better hydrated, had less intestinal inflammation, more rapidly returned to normal proportions of blood leukocytes, and suffered less damage to their intestinal villi. This evidence demonstrates that feeding hLZ-milk at the onset of an intestinal ETEC infection is an effective treatment. Since lysozyme is part of the innate immune system, humans are constantly exposed to it through endogenous secretions including saliva and intestinal mucus, making it an ideal supplementary antimicrobial protein because it poses no risk as an allergen or toxin. For example, breast milk contains high concentrations of lysozyme and breastfeeding is a recommended treatment for diarrhea, however when breastfeeding is not possible oral rehydration therapy is often used. Oral rehydration therapy only addresses some of the symptoms of diarrhea, specifically loss of fluids and electrolytes. Ruminant milk containing hLZ helps the body remedy the infection faster than ruminant milk alone, restoring both the absorptive and barrier functions of the intestine, while providing fluids, electrolytes, and nutrients, making it a more comprehensive treatment for diarrhea than oral rehydration therapy. Based on these results, hLZ-milk from transgenic goats of the Artemis line could provide a safe, sustainable, and direct source of lysozyme-rich milk for communities facing high rates of childhood diarrhea.
